# Prediction of Geometric Characteristics of Laser Cladding Layer Based on Least Squares Support Vector Regression and Crested Porcupine Optimization

**DOI:** 10.3390/mi15070919

**Published:** 2024-07-16

**Authors:** Xiangpan Li, Junfei Xu, Junhua Wang, Yan Lu, Jianhai Han, Bingjing Guo, Tancheng Xie

**Affiliations:** 1 School of Mechanical and Electrical Engineering, Henan University of Science and Technology, Luoyang 471003, China; xiangpanli@haust.edu.cn (X.L.); mecha_xjf@163.com (J.X.); bingjing@haust.edu.cn (B.G.); xietc@haust.edu.cn (T.X.); 2 Henan Intelligent Manufacturing Equipment Engineering Technology Research Center, Luoyang 471003, China; 3 Henan Engineering Laboratory of Intelligent Numerical Control Equipment, Luoyang 471003, China; 4 Henan Collaborative Innovation Center of High-End Bearing, Luoyang 471003, China; 5 School of Materials Science and Engineering, Henan University of Science and Technology, Luoyang 471023, China; luyan@haust.edu.cn

**Keywords:** laser cladding, geometric characteristics, crowned porcupine optimization, least squares support vector regression

## Abstract

The morphology size of laser cladding is a crucial parameter that significantly impacts the quality and performance of the cladding layer. This study proposes a predictive model for the cladding morphology size based on the Least Squares Support Vector Regression (LSSVR) and the Crowned Porcupine Optimization (CPO) algorithm. Specifically, the proposed model takes three key parameters as inputs: laser power, scanning speed, and powder feeding rate, with the width and height of the cladding layer as outputs. To further enhance the predictive accuracy of the LSSVR model, a CPO-based optimization strategy is applied to adjust the penalty factor and kernel parameters. Consequently, the CPO-LSSVR model is established and evaluated against the LSSVR model and the Genetic Algorithm-optimized Backpropagation Neural Network (GA-BP) model in terms of relative error metrics. The experimental results demonstrate that the CPO-LSSVR model can achieve a significantly improved relative error of no more than 2.5%, indicating a substantial enhancement in predictive accuracy compared to other methods and showcasing its superior predictive performance. The high accuracy of the CPO-LSSVR model can effectively guide the selection of laser cladding process parameters and thereby enhance the quality and efficiency of the cladding process.

## 1. Introduction

Laser cladding technology is a surface engineering technology that utilizes a laser as the heat source. This method involves the rapid melting of metal powder and the substrate under the influence of a high-energy laser beam, resulting in the formation of a molten pool. The molten material is then rapidly solidified into a dense and homogeneous metallurgical bonding layer [1]. The laser cladding presents several advantages, such as low dilution rate, reduced work piece deformation, minimized heat-affected zone, and ease of automation. In recent years, laser cladding has gained considerable attention and has found widespread use in diverse fields, including aerospace, automobile, and defense industries [2]. The laser cladding process is a complex physical metallurgical process, and the formation state of the clad layer is influenced by multiple factors. Among these, the cladding morphology is a significant characteristic indicator of laser cladding technology [3]. The quality of the formed cladding can be significantly affected by variations in its width and height. Inappropriate width and height may result in reduced efficiency in the forming process and increased likelihood of thermal stress and material distortion [4,5]. The dimensions of a cladding layer are influenced by various factors, such as laser power, scanning speed, and powder feed rate [6]. Consequently, it is vital to conduct an exploration of the mapping relationship between process parameters and cladding dimensions. This exploration would enable accurate prediction of cladding dimensions at specific parameter inputs, thus facilitating precise regulation of clad dimensions and enhancing forming quality.

Researchers have conducted numerous studies on the prediction of cladding dimensions for laser cladding. Gu et al. [7] and colleagues employed laser cladding technology to produce Fe-Cr-B-Al-Ti overlays aimed at improving the wear resistance of 2Cr13 steel. The research examined how variations in laser scanning velocity and titanium content impact the coatings’ microstructure, dilution levels, microhardness, and overall wear resistance. Gao et al. [8]. formulated a three-dimensional numerical model called the Single-Track Processing Prediction Model (STPPM) for use in laser cladding, leveraging the birth-and-death element approach to anticipate the dimensions of the cladding layer’s width and height during a single-pass laser cladding operation on 316 L stainless steel. The prediction error was less than 6.6%. Li et al. [9] developed a physics-based model for predicting melt-pool geometry and temperature in single-bead directed energy deposition, validated with Ti-6AL-4V tracks on an Optomec LENS system, showing less than 10% error in part height measurements. Chen et al. [10] introduced an advanced 3D multi-physics finite element model that incorporates heat transfer, fluid dynamics, shielding gas pressure, powder temperature elevation, surface tension, and free surface movement. This model offers strong adaptability and can be applied flexibly across various laser cladding systems and materials. The model’s predictions align closely with experimental data, with average discrepancies of 6.05% in width and 4.15% in height. The limitations of the above study are that the laser cladding process is a complex multi-field coupled process, there is a dearth of knowledge regarding the dynamic evolution of the cladding process, and the finite element simulation process relied on numerous over-simplified assumptions, which may have led to potential inaccuracies in the findings. Additionally, the thermodynamic model used in the study is single and not applicable to all scenarios. Moreover, the accuracy and stability of the predictions are heavily reliant on the chosen cell type, boundary conditions, mesh scheme, and other factors. As a result, the accuracy and stability of the prediction are low.

In contrast to finite element methods, machine learning algorithms do not necessitate a comprehensive understanding of intricate physics for identifying the association between process parameters and cladding quality. As a result, the application of machine learning has become increasingly prevalent in the field of additive manufacturing in recent years [11]. Mondal et al. [12] utilized an Artificial Neural Network (ANN) to predict the dimensions of the cladding layer’s width and depth, achieving results that were in close alignment with the experimental outcomes. Gao et al. [13] applied both Random Forest (RF) and Multilayer Perceptron Backpropagation Neural Network (BPNN) to study the correlation between process parameters and the geometric characteristics of single tracks in the laser metal deposition of TC11 alloy. They considered laser power, scanning speed, and powder feed rate as input variables and track width and height as the output. The study’s experimental results demonstrated that the BPNN model had a smaller maximum error than the RF model. Gao et al. [14] used the BPNN model, the Random Forest (RF) algorithm, and the response surface method (RSM) to predict the clad dimensions, respectively, and the results showed that the BPNN model gave the best agreement between the predicted results and the actual values. In summary, machine learning has shown great advantages in the field of additive manufacturing. Previous studies have predominantly used BP neural networks to predict cladding layer dimensions. However, BP neural networks require a high quantity and quality of datasets, while laser cladding is typically a small-sample experiment, making it unsuitable for BP neural networks. Least Squares Support Vector Regression [15] (LSSVR) performs well in small-sample prediction tasks, as it can establish nonlinear relationships between inputs and outputs with fewer data. Therefore, this study uses LSSVR to predict cladding layer dimensions. The penalty factor and kernel parameters of the LSSVR model significantly impact prediction accuracy, and the Crested Porcupine Optimization [16] (CPO) algorithm is used to optimize these parameters, enhancing the prediction accuracy.

This paper proposes a novel approach to predict cladding dimensions with the goal of improving the accuracy of such predictions. Specifically, the proposed method utilizes a Crowned Porcupine Optimization Least Squares Support Vector Regression (CPO-LSSVR) model, which trains and tests the LSSVR model using single cladding size data obtained from orthogonal experiments. Subsequently, the CPO-LSSVR model employs the Crowned Porcupine Optimization algorithm to determine the penalty factor and kernel parameters of the LSSVR model, resulting in a reliable and efficient prediction model that relates the laser cladding process parameters to the cladding dimensions. This study offers valuable reference and guidance for decision-making related to accurate prediction of cladding dimensions, regulation of cladding dimensions, and improvement of forming quality.

## 2. Materials and Methods

### 2.1. Materials and Setup

**Figure 1:**
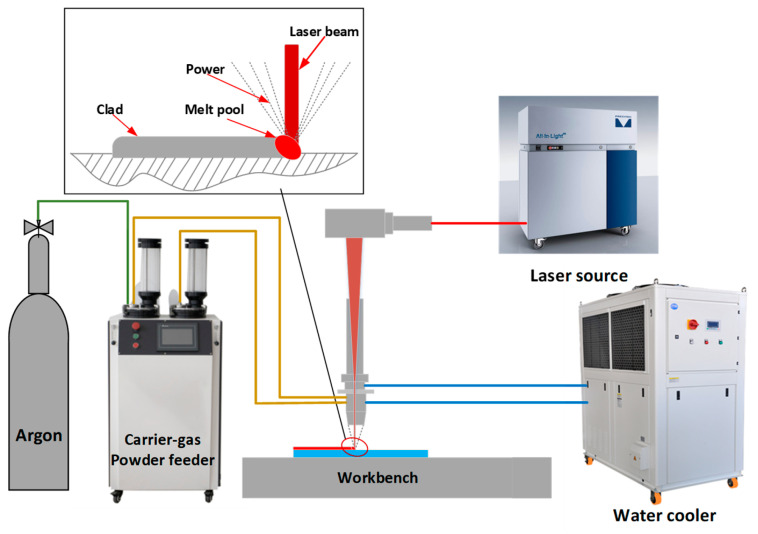
Schematic diagram of the laser cladding system.

All experiments conducted for this research utilized a Laser Cladding and Deposition (LCD) system. As depicted in Figure 1, the LCD system is composed of a laser unit, a powder feeding mechanism, a coaxial powder nozzle, and a cooling apparatus. The laser head is attached to a 3-kilowatt laser source to provide the necessary energy. It operates with a laser spot diameter of 1 mm and a working distance set at 18 mm. Powder is transported to the melt pool by a powder feeder aided by a carrier gas, with Argon serving dual roles as the feed and protective gas at a flow rate of 8 L per minute. To safeguard the equipment, a water-based cooling system is integrated with the laser head to help regulate and reduce its temperature.

In this study, 20 × 10 × 8 mm of 45 steel was used as the substrate. To avoid the influence of repeated processing on the experimental results, only one laser cladding experiment was carried out per substrate. Before the experiment, the substrate surface was sanded and then cleaned of surface impurities using anhydrous ethanol. The 17-4PH is a martensite precipitation hardening stainless steel that exhibits superior strength, hardness, and exceptional corrosion resistance. These advantageous properties have resulted in its widespread utilization in various industrial applications, including aerospace, nuclear power, and medical devices. Its exceptional mechanical and corrosion resistance properties make it an ideal material for critical applications that require high performance and reliability. In this study, 17-4PH powder was used as the cladding material. The particle size distribution of the powder was 75~120 μm, and the chemical composition is shown in Table 1.

### 2.2. Design of Experiments

The utilization of the orthogonal test approach ensures a more uniform distribution of sample data. This technique meets testing design requirements with a reduced number of experiments, enhancing efficiency and reducing costs. In this study, laser power, scanning speed, and powder feed rate are used as input variables, and a predictive model is developed to link these process parameters with the dimensions of the melt layer, with the layer’s width and height serving as output variables. Following the orthogonal experimental methodology, 25 sets of laser cladding experiments were crafted, each with varying levels—five for laser power, scanning speed, and powder feed rate. Table 2 provides detailed information on the experimental design.

## 3. Results

**Figure 2:**
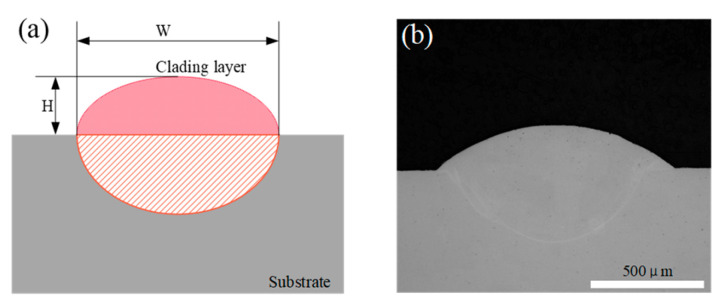
Cross-section of the cladding layer. (**a**) The schematic diagram; (**b**) the sample.

According to the experimental design shown in Table 2, 25 sets of laser cladding experiments were carried out with different process parameters. The cladding was cut using a Wire Cut EDM in a direction perpendicular to the laser scan. After cutting, the cross-section of the cladding was ground with #200, #400, #600, #800, #1000, #1500, #1800, and #2200 grit sandpapers, followed by polishing with a grinding and polishing machine. Finally, it was etched with an etchant composed of 4 g of CuSO_4_, 20 mL of HCl, and 20 mL of H_2_O. The cross-section of the clad layer, after the grinding and polishing process, was photographed using an optical microscope, and the shape of the clad layer is shown in Figure 2.

ImageJ software (ImageJ2) was used to measure the width and height of the cladding layer to obtain the width and height of the cladding layer for different process parameters. The results of the orthogonal experiment are shown in Table 3. P, V, and f in Table 3 represent the three process parameters of laser power, scanning speed, and powder feeding rate, respectively. W and H represent the width and height of the cladding layer, respectively.

To investigate the effect of different process parameters on the width and height of the cladding layer, an analysis of variance (ANOVA) was carried out on the experimental results. The ANOVA conducted on the width of the cladding layer has revealed that the laser power parameter in the laser cladding process exerts the most significant influence on the clad width. This is followed by the scanning speed parameter, while the powder feed parameter demonstrates the least impact on the width. A multivariate nonlinear fit was carried out to investigate the relationship between the process parameters and the cladding width. The process parameters were considered as independent variables, and the cladding width was considered as the dependent variable. This analysis was performed using the nonlinear least squares–McQuart method for 25 sets of experiments. The objective function of the cladding width and the laser cladding process parameters was obtained, taking into account the nonlinear nature of the relationship between these variables. The objective function is shown below:

(1) $$W=7.203\left(P^{1.325}\times V^{-0.872}\times f^{0.473}\right)-7.913$$

As can be seen from objective function 1, the cladding width increases with increasing laser power and powder feed, and the scanning speed increases as the cladding width decreases.

The ANOVA conducted on the height of the cladding layer has revealed that the scanning speed parameter in the laser cladding process exerts the most significant influence on the clad width. This is followed by the laser power parameter, while the powder feed parameter demonstrates the least impact on the width. Similarly, using the process parameters as the independent variables and the height of the cladding layer as the dependent variable, a multivariate nonlinear fit of the experimental results was performed to obtain the objective function of the cladding height versus the laser cladding process parameters as follows:

(2) $$H=1.667\left(P^{-0.743}\times V^{-1.115}\times f^{0.328}\right)+3.163$$

As can be seen from the objective function, the cladding height decreases as the laser power and scanning speed increase, and the cladding height increases as the powder feed volume increases.

## 4. Prediction Modeling

### 4.1. LSSVR Prediction Model

Least Squares Support Vector Regression (LSSVR) is an extension of the Support Vector Regression (SVR) algorithm [17]. By minimizing a quadratic programming problem, LSSVR finds the optimal solution efficiently, effectively handling nonlinear regression problems while reducing algorithm complexity. Compared to the SVR algorithm, LSSVR significantly improves computational speed and convergence accuracy [18]. The relationship between the laser cladding process parameters and the size of the cladding layer is nonlinear and represents a typical small-sample scenario, making the LSSVR model highly suitable for predicting the cladding layer in laser cladding processes.

In a given training set containing *N* points ${\left\{x_{i},y_{i}\right\}}^{N}$, $x_{i}$ is inputs, and $y_{i}$ is the output. The objective function can be expressed as follows:

(3) $$f(x)=\omega^{T}\varphi (x_{i})+b$$

where ω is the weight vector; $x_{i}$ is the input vector; $\varphi (x_{i})$ is the nonlinear function that maps the input matrix to a higher-dimensional feature space; and *b* is the bias term.

The optimization problem for LSSVR is

(4) $$\begin{array}{l} \min J(w,\xi )=\frac{1}{2}w^{T}w+\frac{1}{2}C\sum_{i=1}^{l}\xi_{i}^{2}\\ S.t.y_{i}=\omega^{T}\varphi (x_{i})+b+\xi_{i}\;(i=1,2,\cdots ,l) \end{array}$$

where $C\in R^{+}$ is the penalty coefficient; $y_{i}$ is the output vector; *J* is the loss function; and $\xi_{i}\in R$ is the slack variable;

The constraint conditions are

(5) $$y_{i}=\omega^{T}\varphi (x_{i})+b+\xi_{i}\;i=1,2,\cdots ,l$$

Through its dual form, the Lagrangian function is established based on the objective function and the constraint conditions:

(6) $$L(\omega ,b,\xi ,\alpha )=\frac{1}{2}\omega^{T}\omega +\frac{1}{2}C\sum_{i=1}^{l}\xi_{i}^{2}-\sum_{i=1}^{l}\alpha_{i}[\omega^{T}\varphi (x_{i})-y_{i}+b+\xi_{i}]$$

where $\alpha_{i}$ is the Lagrange multiplier, $\alpha_{i}>0$.

According to the Karush–Kuhn–Tucker (KKT) conditions, taking the partial derivatives with respect to ω, $\xi_{i}$, *b*, and $\alpha_{i}$ and setting them to zero, we have

(7) $$\left\{\begin{array}{c} \frac{\partial L}{\partial \omega }=0\\ \frac{\partial L}{\partial b}=0\\ \frac{\partial L}{\partial \xi_{i}}=0\\ \frac{\partial L}{\partial \alpha_{i}}=0 \end{array}\right.$$

We obtain the following expressions:

(8) $$\left\{\begin{array}{c} \omega -\sum_{i=1}^{l}\alpha_{i}\varphi (x_{i})=0\\ \sum_{i=1}^{l}\alpha_{i}=0\\ \alpha_{i}=C\xi_{i}\\ \omega^{T}\varphi (x_{i})+b+\xi_{i}-y_{i}=0 \end{array}\right.$$

Using the method of kernel functions for calculation, the kernel function expression is as follows:

(9) $$K(x,x_{i})=\varphi^{T}(x)\varphi (x_{i})$$

Finally, we obtain the following matrix:

(10) $$\left[\begin{array}{cc} 0 & I^{T}\\ I & \Omega +C^{-1}E \end{array}\right]\times \left[\begin{array}{c} b\\ \alpha \end{array}\right]=\left[\begin{array}{c} 0\\ y \end{array}\right]$$

where $I={\left[1,1,\cdots ,1\right]}^{T}$; *E* is the identity matrix of dimension l×l; $\alpha ={\left[\alpha_{1},\alpha_{2},\cdots ,\alpha_{l}\right]}^{T}$; and $y={\left[y_{1},y_{2},\cdots ,y_{l}\right]}^{T}$; $\Omega_{ij}=\varphi (x_{i})\cdot \varphi (x_{j})=K(x_{i},x_{j})$ is a kernel function that satisfies the Mercer condition.

In this paper, we select the Gaussian RBF function, which has fewer parameters and strong learning capability, as the kernel function for the LSSVR model. By solving the equation above, we finally obtain the decision function of LSSVR as

(11) $$f(x)=\sum_{i=1}^{l}a_{i}\times \exp(-\frac{\left\|x_{i}-x_{j}\right\|}{2\sigma^{2}})+b$$

From the 25 sets of test data, 5 sets were randomly selected as the testing set and the remaining 20 sets as the training set. Firstly, the data from the training set were imported into the LSSVR. Since the experimental data are not of uniform dimension and the values of different parameters vary greatly from one another, the data need to be normalized and all data converted to between [0, 1], which can speed up the convergence and improve the prediction accuracy. The normalization equation is shown as follows:

(12) $$X^{\prime }=\frac{X-X_{\min}}{X_{\max}-X_{\min}}$$

where X is the original data; $X_{\max}$, $X_{\min}$ are the maximum and minimum values of the original data, respectively; and $X^{\prime }$ is the normalized data.

### 4.2. Crested Porcupine Optimizer (CPO) Algorithm for optimizing Least Squares Support Vector Regression (LSSVR)

In LSSVR, hyperparameters significantly influence model prediction performance. This study employs an RBF kernel function to construct the LSSVR model, where the penalty factor C and kernel parameter σ exert the greatest influence on prediction performance [19]. To enhance prediction accuracy and reliability, this study utilizes the CPO algorithm to optimize the parameters of LSSVR.

#### 4.2.1. Crested Porcupine Optimizer Algorithm

The CPO algorithm is a novel metaheuristic optimization technique that mimics four defense behaviors of the crested porcupine (visual, auditory, olfactory, and physical attacks) to solve optimization problems. It possesses strong global search capabilities and features rapid convergence.

The CPO algorithm, like other metaheuristic swarm algorithms, initiates the search process from an initial set of individuals. For each individual *i*, a random position $X_{i}$ is generated within the search space, expressed mathematically as follows:

(13) $$X_{i}=L+r\times (U-L)\;\;i=1,2,\cdots ,N$$

where *N* is the population size; *r* is a uniformly distributed random number between 0 and 1; and *L* and *U* are the lower and upper bounds of the search range, respectively.

To enhance the algorithm’s convergence speed while maintaining population diversity, the Cycle Population Reduction (CPR) technique periodically increases or decreases the population size according to a predefined pattern. The mathematical expression is

(14) $$N(t)=N_{\min}+(N^{\prime }-N_{\min})\times (1-\frac{t\%T_{\max}}{T_{\max}})$$

where *N*(*t*) is the population size at the current iteration t; *N*_min_ is the minimum population size; $N^{\prime }$ is the initial population size; *T*_max_ is the maximum number of iterations for the algorithm; *t* is the current iteration number; % denotes the modulo operation used to apply adjustments periodically.

The purpose of the visual strategy is to simulate the behavior of a crested porcupine when it detects a predator. In the algorithm, this corresponds to the behavior of individuals moving towards the global best solution. The mathematical expression is

(15) $$X_{i}^{t+1}=X_{CP}^{t}+\tau_{1}\times (2\times \tau_{2}\times (X_{CP}^{t}-y_{i}^{t}))$$

where $X_{i}^{t+1}$ represents the position of the *i*th individual in the next-generation iteration; $X_{CP}^{t}$ represents the position of the current global best solution, i.e., the position of the best individual found at the current iteration *t*; $\tau_{1}$ is a random number based on a normal distribution, used to simulate randomness and enhance the algorithm’s exploration capability; $\tau_{2}$ is a random number in the interval [0, 1] used to adjust the intensity of exploration; $X_{CP}^{t}-y_{i}^{t}$ represents the difference between the current global optimal solution and the position of the current individual *i*; $y_{i}^{t}$ represents the midpoint position between individual *i* and a random individual at iteration *t*, used to simulate the position of the predator.

The purpose of the sound strategy is to simulate the behavior of crested porcupines emitting sounds when a predator approaches. In the algorithm, this represents the update of the individual’s position. The mathematical expression is

(16) $$X_{i}^{t+1}=(1-U_{1})\times X_{i}^{t}+U_{1}\times (y+\tau_{3}\times (X_{r1}^{t}-X_{r2}^{t}))$$

where $X_{i}^{t+1}$ represents the position of the *i*th individual in the next iteration; $X_{i}^{t}$ represents the position of the *i*th individual at the current iteration *t*; $U_{1}$ is a random vector with elements valued at 0 or 1, used to simulate whether the crested porcupine emits a threatening sound to the predator and the intensity of the sound; *y* represents the position of the predator, usually calculated from the positions of two random individuals in the current population; $\tau_{3}$ is a random value in the interval [0, 1] used to control the step size of the predator’s movement; $X_{r1}^{t}$ and $X_{r2}^{t}$ represent the positions of two different randomly selected individuals from the population at the current iteration t.

The scent strategy simulates the behavior of crested porcupines releasing a scent to repel predators. In the algorithm, this is represented by the movement and update of individuals in the search space to explore new solutions and avoid local optima. In this way, the algorithm can gradually approach the global optimum while maintaining population diversity. The mathematical expression is

(17) $$X_{i}^{t+1}=(1-U_{1})\times X_{i}^{t}+U_{1}\times (X_{r_{1}^{\prime }}+S_{i}^{\prime }\times (X_{r_{2}^{\prime }}-X_{r_{3}^{\prime }}))-\tau_{3}\times \delta \times \gamma_{t}\times S_{i}^{\prime }$$

where $X_{i}^{t+1}$ represents the position of the *i*th individual at the next iteration *t* + 1; $X_{i}^{t}$ represents the position of the *i*th individual at the current iteration *t*; $U_{1}$ is a random vector with elements valued at 0 or 1, used to simulate whether the crested porcupine releases a scent. When the value is 1, the individual participates in the scent strategy update; when the value is 0, it does not participate in the scent strategy update; $X_{r_{1}^{\prime }}$ represents the position of a randomly selected individual used to calculate the new position; $S_{i}^{\prime }$ is the scent diffusion factor used to control the intensity of the position update in the scent strategy; $X_{r_{2}^{\prime }}$ and $X_{r_{3}^{\prime }}$ represent the positions of two other randomly selected individuals in the search space, used along with $X_{r_{1}^{\prime }}$ to calculate the direction and intensity of the scent diffusion; $\tau_{3}$ is a random value in the interval [0, 1] used to control the influence of randomness in the scent strategy; δ represents a parameter that controls the search direction, used to adjust the movement direction of the individual in the search space; $\gamma_{t}$ is the defense factor, a time-varying function used to simulate defensive behaviors at different points in time. The mathematical expression is as follows:

(18) $$\gamma_{t}=2\times rand\times {\left(1-\frac{t}{t_{\max}}\right)}^{\frac{1}{t_{\max}}}$$

where $t_{\max}$ is the maximum number of iterations.

The physical attack strategy simulates the defensive behavior of crested porcupines, allowing the algorithm to conduct more focused and target-oriented searches during the search process. This strategy is particularly suitable for the later stages of the algorithm, when more refined searches are needed in identified promising areas. The mathematical expression is

(19) $$X_{i}^{t+1}=X_{CP}^{t}+\alpha \times (1-\tau_{4})\times (X_{CP}^{t}+X_{i}^{t})+\tau_{4}\times F_{CP}^{t}$$

where $X_{i}^{t+1}$ represents the position of the *i*th individual at the next iteration *t* + 1; $X_{CP}^{t}$ represents the position of the global optimal solution at the current iteration *t*; α is the convergence factor used to control the speed at which the algorithm converges to the optimal solution; $\tau_{4}$ is a random value in the interval [0, 1] used to introduce randomness in the physical attack strategy; $F_{CP}^{t}$ represents the average force exerted by the global optimal solution $X_{CP}^{t}$ on predators at the current iteration *t*. This can be computed based on the principles of elastic collision:

(20) $$F_{CP}^{t}=\tau_{5}\times \alpha_{i}\times (X_{CP}^{t}+X_{i}^{t})$$

where $\tau_{5}$ represents another random number within the interval [0, 1], used to further control the strength of the force; $\alpha_{i}$ is a parameter related to the importance of the *i*th individual.

**Figure 3:**
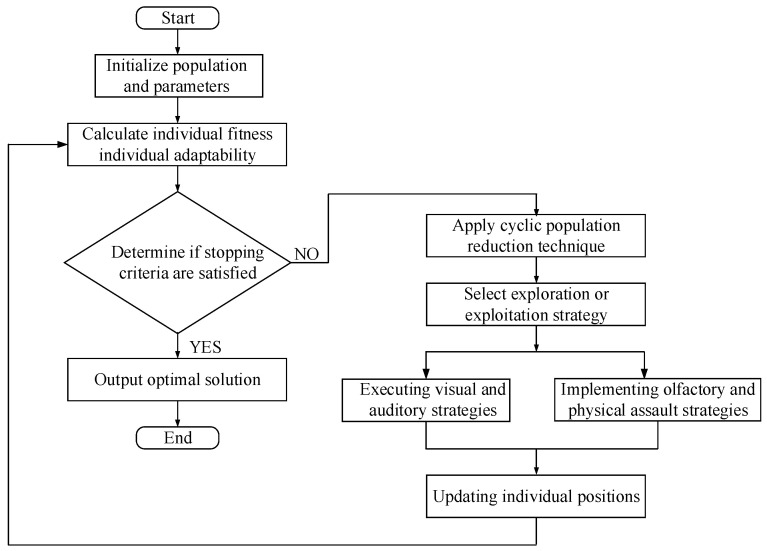
Crested porcupine optimization algorithm flowchart.

The flowchart of the Crested Porcupine Optimization (CPO) algorithm is shown in Figure 3:

#### 4.2.2. CPO-LSSVR

**Figure 4:**
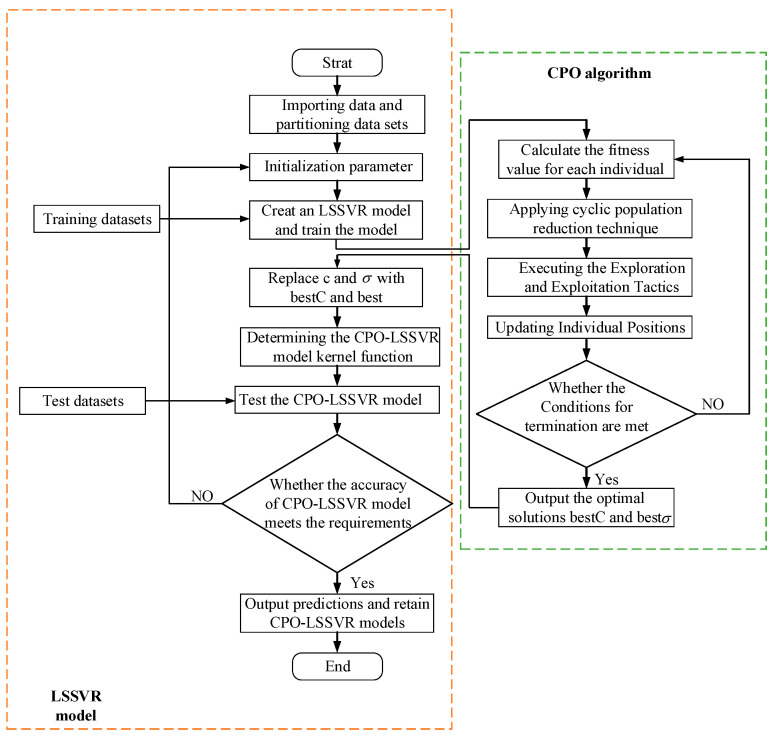
Flowchart of CPO algorithm optimization for LSSVR model.

The CPO algorithm is used to optimize the penalty factor C and kernel parameter σ of the LSSVR model to improve prediction accuracy. The process is shown in Figure 4.

Step 1: Input process parameters into the model, use deposition layer width and height as output data inputs to the model, and divide into training and testing sets;

Step 2: Set initial parameters, including the search range for penalty factor c and kernel parameter σ, and randomly generate a population;

Step 3: Build the LSSVR model and train it using the training set;

Step 4: Substitute the optimization results of each individual into the LSSVR model. Calculate the mean squared error between the predicted values obtained from different LSSVR model training processes and the experimental values. Use this as the fitness function for the CPO algorithm and compute the fitness of all individuals;

Step 5: Apply cyclic population reduction techniques to periodically reduce the size of the population;

Step 6: Execute exploration and exploitation strategies, updating individual positions;

Step 7: Determine if the termination condition is met. If satisfied, output the optimal penalty factor *bestC* and optimal kernel parameter bestσ. If not satisfied, proceed to Step 4;

Step 8: Replace the LSSVR model’s c and σ with *bestC* and bestσ;

Step 9: Test the CPO-LSSVR model using the test dataset.

## 5. Results and Discussion

### 5.1. LSSVR Model Prediction Results

The training data and test data are trained and tested using the constructed LSSVR model. This study conducted a total of 25 experiments, randomly selecting 20 of them as the training set, with the remaining 5 serving as the test set. The true height and width values of the test set are compared with the predicted height and width values to verify the accuracy of the model. Relative error is defined as follows:

(21) $$\Delta =\frac{X-X^{\prime }}{X}\times 100\%$$

where X is the actual data value of the test set, and $X^{\prime }$ is the predicted value of the model.

Table 4 shows the actual values of the width and height of the test set compared to the predicted values of the LSSVR model. Specifically, the symbols “W” and “W1” are employed to indicate the actual and predicted values of the width, respectively, while “H” and “H1” denote the measured and predicted values of the height, respectively. Furthermore, the symbols “Δ1” and “Δ2” are adopted to represent the relative error between the predicted and measured values of the width and height, respectively. As can be seen from Table 4, the relative error in width is within 10.82% and the relative error in height is less than 11.11%.

**Figure 5:**
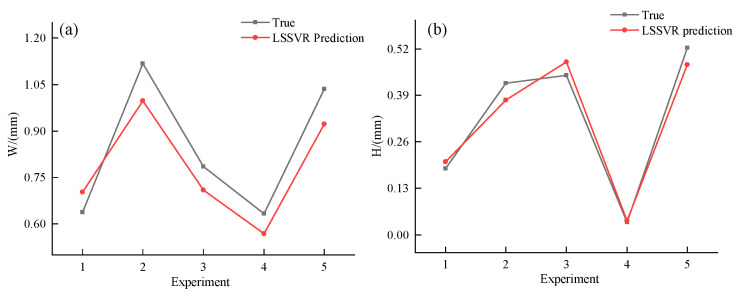
Comparison of the actual values of the test set with the predicted values of LSSVR. (**a**) Graph of actual width values compared to predicted width values. (**b**) Graph of actual height values compared to predicted height values.

In order to visually convey the extent of variance between the predicted and true values, it is often beneficial to utilize a graphical representation of the data in the form of an image. This approach provides a more intuitive and easily interpretable depiction of the degree to which the predictions deviate from the actual values. The results are shown in Figure 5. The solid red line represents the actual values and the solid blue line represents the predicted values from the LSSVR model; Figure 5a shows the actual values of width compared to the model predictions and Figure 5b indicates the actual values of height compared to the model predictions.

According to Figure 5, the developed LSSVR model has some errors in the prediction of the cladding layer size. An analysis of the potential sources of error indicates that the LSSVR model may not have been optimized, with the penalty factor C and the kernel parameter σ possibly not being the best parameters, which could affect the prediction accuracy.

### 5.2. Comparison of Prediction Results from Different Models

**Figure 6:**
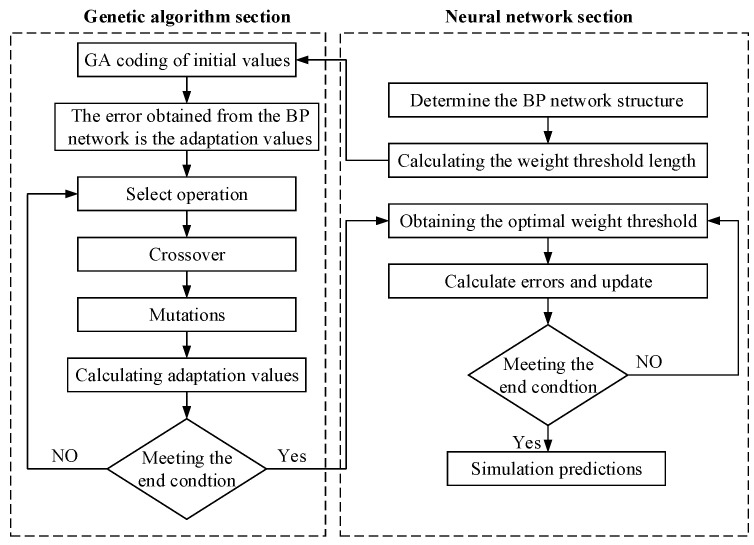
Diagram of the GA-BP process.

Optimize the parameters C and A of the LSSVR model using the CPO algorithm. Use the constructed CPO-LSSVR overlay size prediction model to predict the width and height of the overlay. Backpropagation Neural Networks (BPNNs) have been widely applied in regression prediction problems within the field of additive manufacturing, achieving satisfactory results. In this study, the BPNN is selected as the control group. To objectively compare the predictive accuracy of the CPO-LSSVR model, a Genetic Algorithm [20,21] is employed to determine the optimal weights and thresholds for the BPNN [22]. A Genetic Algorithm-optimized BP neural network prediction model is constructed, as depicted in Figure 6.

Train the LSSVR model, CPO-LSSVR model, and GA-BP model separately using the training set, and evaluate these models using the test set. Table 5 compares the overlay width predictions between actual values and predictions from different models. Here, W represents the actual values, W1 represents predictions from the LSSVR model, W2 represents predictions from the CPO-LSSVR model, and W3 represents predictions from the GA-BP model. Δ1 denotes the relative error between predictions of the LSSVR model and actual values, Δ2 denotes the relative error for the CPO-LSSVR model, and Δ3 denotes the relative error for the GA-BP model.

**Figure 7:**
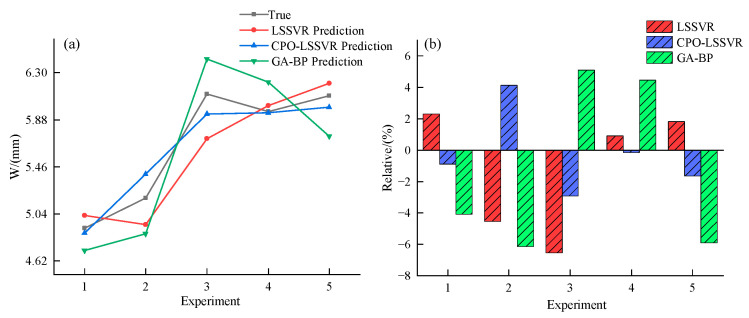
Comparison of overlay width prediction performance among different models. (**a**) Comparison of predicted values from different models. (**b**) Comparison of relative errors among different models.

To visually illustrate the predictive performance of the models, the data were subjected to visual analysis, as shown in Figure 7.

As shown in Table 5 and Figure 7, the predicted values of the CPO-LSSVR model are closer to the actual values, with smaller relative errors. Specifically, the average relative error of the LSSVR model is 3.226%, the CPO-LSSVR model is 1.956%, and the GA-BP model is 5.138%. The average relative error is defined as follows:

(22) $$\eta =\frac{1}{n}\sum_{i=1}^{n}\left|\Delta_{i}\right|$$

where n is the number of experiments in the test set and i is the experiment number.

Similarly, the overlay height predictions were compared, and the results are shown in Table 6. Here, H represents the actual values, H1 represents predictions from the LSSVR model, H2 represents predictions from the CPO-LSSVR model, and H3 represents predictions from the GA-BP model. Δ4 denotes the relative error between predictions of the LSSVR model and actual values, Δ5 denotes the relative error for the CPO-LSSVR model, and Δ6 denotes the relative error for the GA-BP model.

**Figure 8:**
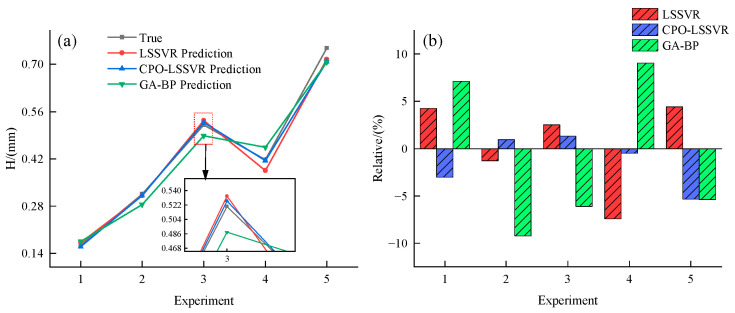
Comparison of overlay height prediction performance among different models. (**a**) Comparison of predicted values from different models. (**b**) Comparison of relative errors among different models.

The data were also visualized, as shown in Figure 8.

As shown in Table 6 and Figure 8, the predicted values of the CPO-LSSVR model are closer to the actual values, with higher prediction accuracy. Specifically, the average relative error of the LSSVR model is 3.972%, the CPO-LSSVR model is 2.231%, and the GA-BP model is 7.314%.

In summary, the CPO-LSSVR model has the highest prediction accuracy and the smallest relative error, making it more capable of accurately describing the nonlinear relationship between process parameters and overlay dimensions. The CPO-LSSVR model can achieve precise predictions of overlay dimensions, which is significant for guiding subsequent analyses and process decisions.

## 6. Conclusions

The main findings and conclusions of this study are as follows:

(1) In this study, the CPO algorithm was used to optimize the penalty factor and kernel parameters of the LSSVR model. The CPO-LSSVR model was utilized to predict the dimensions of the overlay, the results indicate that the CPO-LSSVR model achieved a relative prediction error of less than 2.3%.

(2) In this study, the Backpropagation Neural Network (BPNN), widely used in the field of additive manufacturing, was employed as a control group to establish a Genetic Algorithm (GA)-optimized BPNN control predictive model. The results indicated that the CPO-LSSVR model’s predictive accuracy is higher than that of the GA-BP model. The CPO-LSSVR can reliably and accurately predict the dimensions of the deposition layer, providing effective guidance for improving the formation accuracy of the deposition layer and optimizing process parameters.

## Figures and Tables

**Table 1:** Chemical composition of 17-4PH.

Element	C	Mn	Si	S	P	Cr	Ni	Cu	Nb
Wt%	≤0.07	≤1.0	≤1.0	≤0.025	≤0.035	15.0–17.0	3.0–5.0	3.0–5.0	0.15–0.45

**Table 2:** Design of experiments.

Process Parameters (Symbol, Unit)	Value
Laser power (P, W)	120, 160, 200, 240, 280
Scan speed (v, mm/s)	2, 4, 6, 8, 10
Powder feed rate (f, g/min)	2.6, 5.2, 7.8, 10.4, 13
Argon gas flux (Q, L/min)	8
laser spot diameter (d, mm)	1

**Table 3:** Orthogonal experiment results.

NO.	*P*/(W)	*V*/(mm·s^−1^)	*f*/(g·min^−1^)	*W*/mm	*H*/mm
1	240.00	8	5	0.790	0.174
2	200	2	4	0.926	0.501
3	200	10	5	0.615	0.157
4	200	6	2	0.638	0.072
5	200	8	1	0.695	0.059
6	280	6	5	0.935	0.296
7	120	8	3	0.440	0.031
8	240	4	2	0.826	0.159
9	240	6	1	0.802	0.072
10	120	6	4	0.605	0.095
11	280	10	3	0.851	0.112
12	240	10	4	0.730	0.114
13	160	8	2	0.456	0.049
14	160	8	4	0.671	0.173
15	280	8	1	0.984	0.123
16	160	6	3	0.534	0.076
17	280	8	4	0.892	0.202
18	120	2	1	0.691	0.126
19	120	10	2	0.406	0.069
20	120	4	5	0.365	0.066
21	200	4	3	0.638	0.186
22	280	2	2	1.118	0.424
23	160	2	5	0.786	0.446
24	160	10	1	0.633	0.035
25	240	2	3	1.035	0.523

**Table 4:** Data comparison between test values of test set and predicted values of LSSVR model.

NO.	W	W1	Δ1/%	H	H1	Δ2/%
1	0.638	0.703	−10.19	0.1856	0.204	−9.91
2	1.118	0.998	10.733	0.4236	0.377	11
3	0.786	0.709	9.8	0.4456	0.483	−8.39
4	0.633	0.568	10.27	0.0351	0.039	−11.11
5	1.035	0.923	10.82	0.5227	0.476	8.93

**Table 5:** Comparison of actual and predicted deposition layer widths for each model test set.

NO.	W	W1	W2	W3	Δ1/%	Δ2/%	Δ3/%
1	4.913	5.026	4.869	4.712	2.3	−0.896	−4.091
2	5.181	4.945	5.395	4.863	−4.555	4.13	−6.137
3	6.11	5.71	5.931	6.422	−6.547	−2.93	5.106
4	5.953	6.007	5.943	6.218	0.907	−0.168	4.451
5	6.094	6.205	5.993	5.734	1.821	−1.657	−5.907

**Table 6:** Comparison of actual and predicted deposition layer height for each model test set.

NO.	H	H1	H2	H3	Δ4/%	Δ5/%	Δ6/%
1	0.165	0.172	0.16	0.176	4.242	−3.03	7.123
2	0.315	0.311	0.312	0.285	−1.27	0.952	−9.237
3	0.52	0.533	0.527	0.488	2.5	1.346	−6.115
4	0.417	0.386	0.415	0.454	−7.434	−0.48	9.02
5	0.748	0.715	0.708	0.707	4.412	−5.348	−5.375

## Data Availability

The original contributions presented in the study are included in the article, further inquiries can be directed to the corresponding author.
